# Processing Incongruity for Mental Events in Comics: Contours of Character Inferences

**DOI:** 10.1080/10926488.2024.2417215

**Published:** 2024-12-31

**Authors:** Bien Klomberg, Klavdiia Fadeeva, Joost Schilperoord, Neil Cohn

**Affiliations:** Tilburg University

## Abstract

Visual narratives, like comics, at times show depictions of characters’ imagination, dreams, or flashbacks, which seem incongruent with the ongoing primary narrative. Such “domain constructions” thus integrate an auxiliary domain (e.g. a dream) within the primary domain (the expected, physical storyworld), and may require readers to resolve seemingly non-co-referential figures as co-referential (e.g. when a character’s dream shows that character as an animal). In three self-paced reading experiments, we investigate the processing and understanding of single vs. multiple domains in sequences with co-reference issues (Exp. 1) and whether graphic cues facilitate such domain switches (Exp. 2 and 3). Domain switches incurred greater updating costs but were comprehensible, with greater similarity across panels predicting faster processing, and comic reading experience affecting viewing times. The successful integration of fantasized agents which seem to lack co-reference implies that visual narrative comprehension goes beyond event and scene perception alone, but also involves proficiency in conventional constructions related to perspective-taking and inferencing.

Visual narratives, like comics, sometimes show events that have to be understood as not *actually* taking place at that place and/or moment in the storyworld, like flashbacks, hallucinations, and dreams. On the surface, such events seem incongruous with the ongoing narrative until the reader understands these as taking place in someone’s *mind*. Consider Figure 1: panels 1 and 2 show Suzy berating Calvin for not working on their school project and panel 3 shows her yelling at him. The final panel then shows an alien yelling unfamiliar words at a spaceman figure, a scene that differs significantly from the prior characters and scene. However, these divergent figures can make sense when they are seen as part of Calvin’s thoughts (Maier & Bimpikou, 2019). Calvin sees Suzy as an incomprehensible, outer world creature. In Calvin’s mental world, he appears as a spaceman and Suzy as an alien, an interpretation that invites co-reference between these figures. These sequences thus present some incongruity, and to make sense of them, readers should establish co-reference between seemingly distinct appearances. This paper investigates this process of construing continuity out of incongruity, and what features support this resolution.

**Figure 1.**
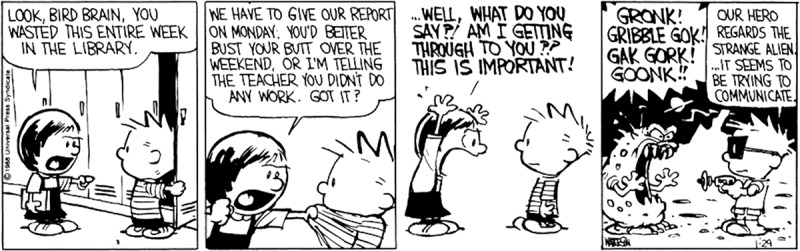
A sequence integrating a primary narrative with an additional context that visualizes a character’s (private) mental experience; sequence from *Calvin & Hobbes* by Bill Watterson © universal press syndicate.

Visual incongruities motivate additional meaning-making when they are recognized by the comprehender as intentional, rather than an occasional mistake (Schilperoord, 2018). Readers recognize that the material diverges from the expectations that they had built up in a cognitive reference model evoked by the scene, which leads to the process of *incongruity resolution*, i.e., finding an explanation that makes sense of the initial incongruity (Forabosco, 2008; Schilperoord, 2018). For Figure 1, panel 4’s entities differ from what would be expected based on the rest of the sequence, prompting readers to try to resolve the sequence. When the image is understood as a representation of a character’s thoughts (a mental world where imagining figures with different appearances is possible), it allows readers to infer co-reference between visually distinct figures. By inferring co-reference, the incongruity is resolved. This resolution thus gives insight into a character’s internal world, and may make a sequence more humorous.

Figure 1’s resolution process illustrates a *domain construction*: a visual incongruity where the resolution requires readers to understand two conceptual *domains* within the sequence. Domains refer to cognitive representations of knowledge (Clausner et al., 1999; Langacker, 1987); in our case, the background knowledge related to types of events. Despite different frameworks, theories of discourse (Abusch & Rooth, 2022; Maier & Bimpikou, 2019), focalization (Forceville, 2023; Horstkotte & Pedri, 2022), perspective taking (Mikkonen, 2017), and film narratives (Luchoomun, 2012) all seem to maintain a similar distinction between events assumed to occur in the physical storyworld (accessible to all characters) and events assumed to not actually occur (typically private to a single character), which are attributed to characters’ imagination, hallucinations, dreams, or memories. We describe this distinction as a *primary domain*, i.e., the world with physically occurring events perceivable to all characters, and an *auxiliary domain*, i.e., events that take place in a mental or “unreal” world, usually (private) thoughts or other mental experiences. How the auxiliary domain relates to the primary domain depends on the inferred nature of the auxiliary domain, e.g., whether it is understood as imagination, a dream, memory, etc. (Klomberg et al., 2024).

Domain constructions may necessitate inferences of co-reference across entities that initially appear distinct but make sense once the divergent appearance is resolved as a representation within an auxiliary domain (Klomberg et al., 2024). In Figure 1, readers can infer that Suzy appears as an alien when they understand that the events are part of Calvin’s imagination (an auxiliary domain). Readers thus need to resolve these appearances as co-referential, despite the differences in form. This resolution typically includes what type of action the experiencer (i.e., the one having the mental experience) is involved in (Klomberg et al., 2023, 2024). For instance, incongruous appearances can be explained as part of an experiencer’s dream, hallucination/imagination, or their memories (e.g., the characters looking much younger). Since the divergent version of the character is valid in an auxiliary domain, we refer to these appearances as *auxiliary identities.*

While prior work has theorized the meaning-making behind these domain constructions (Abusch & Rooth, 2022; Bimpikou, 2018; Maier & Bimpikou, 2019) and their graphic and structural cues (Horstkotte & Pedri, 2022; Klomberg et al., 2024; Mikkonen, 2017), to our knowledge no study has yet empirically tested how readers process these sequences. In general, theories of visual narrative comprehension (Cohn, 2020b; Loschky et al., 2020) describe how readers build up a mental representation of the ongoing narrative, which gets updated with each incoming piece of information as long as the information is sufficiently congruous.

More incongruent information requires longer updating processes (Huff et al., 2020; Hutson et al., 2018; Manfredi et al., 2017, 2018), but can be integrated cohesively. For instance, bridging inferences (sequences that require readers to infer a (missing) key event) demand more effortful processing but are still relatively comprehensible (Cohn & Kutas, 2015; Klomberg & Cohn, 2022). Recent work also showed that processing bridging inferences is not as effortful as processing novel/unrelated events, and that only novel events created robust event boundaries (Brich et al., 2024). This supports that bridging inferences lead to mapping/updating information across panels (rather than constructing a new mental representation), and that such sequences are still perceived as showing one event. Domain constructions similarly present incongruity that can be resolved through inferencing (i.e., constructing an auxiliary domain), which motivates the assumption that readers may also be able to integrate auxiliary domain panels into a coherent mental representation of the ongoing narrative.

The current work sets out to investigate the processing of auxiliary identities in narrative sequences, as in Figure 1 Similar to more incongruous information and other inferential narrative techniques (Brich et al., 2024; Cohn & Kutas, 2015; Cohn & Wittenberg, 2015; Klomberg & Cohn, 2022), we expect auxiliary identities to elicit extended updating but still be understood. During that updating, readers would need to construct an auxiliary domain for these sequences and infer relations of co-reference across primary and auxiliary domain figures. Prior work has posited that such meaning making would be supported by graphic cues (Horstkotte & Pedri, 2022; Klomberg et al., 2024; Mikkonen, 2017). For example, in Figure 1, similarity exists in the composition and contours of the characters’ figures across panels 3 and 4, with Suzy/the alien and Calvin/the spaceman appearing in roughly similar postures. Such similarity across panels may facilitate incongruity resolution, because it seems to draw a parallel between primary and auxiliary domain events. We speculate that such similarity could be intentional to support readers in resolving auxiliary identities, with noticeable similarity helping readers to infer co-referential relations between primary and auxiliary identities.

To explore the processing and properties of domain construction sequences, we conducted three self-paced viewing experiments using comics. Experiment 1 compares sequences with and without auxiliary domains. Experiment 2 tests the effects of including visual similarity for auxiliary identities, and Experiment 3 measures the strength of this visual similarity across panels, and to what extent stronger visual similarity might lead to stronger effects. Data from all experiments are openly available in an online data repository (https://doi.org/10.34894/8OHFN5).

## Experiment 1

To investigate how readers perceive auxiliary identities, the first experiment compares the processing and comprehensibility of sequences with and without a domain switch. Moreover, we investigate the effect of the type of storyworld domain that is shown, by manipulating the direction of the domain switch (from the primary domain to the auxiliary domain and vice versa) and the domain depicted in the single-domain sequences (a sequence showing only primary domain entities vs. only auxiliary domain entities). In line with theories of visual narrative comprehension (Cohn, 2020b; Loschky et al., 2020) and experimental findings for visual sequences with bridging inferences (Brich et al., 2024; Hutson et al., 2018; Klomberg & Cohn, 2022; Magliano et al., 2017), we expect sequences with domain switches to be more effortful to process than those without, because sequences with multiple domains require the process of incongruity resolution to be understood. Despite longer viewing times, we still expect these sequences to be comprehensible.

### Methods

#### Stimuli

**Figure 2.**
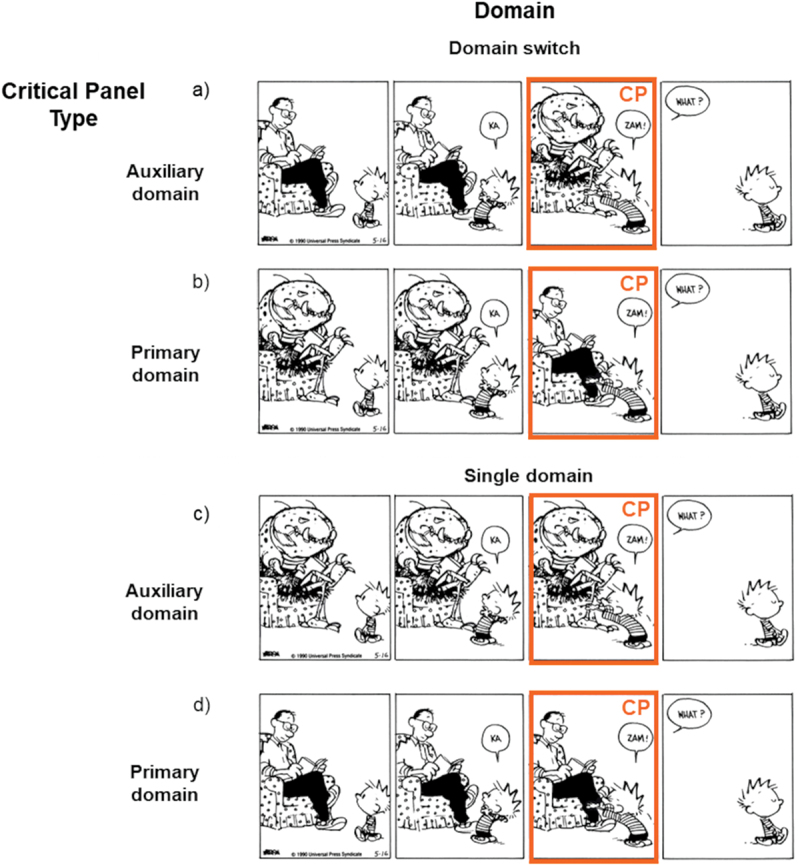
Example stimuli of the 2 × 2 design, showing incongruous sequences with a switch into a) an auxiliary domain, b) a primary domain, and congruous sequences showing c) only auxiliary domain events, and d) only primary domain events; figures slightly adapted from *Calvin & hobbes* by Bill Watterson © universal press syndicate.

We selected 24 *Calvin & Hobbes* comics with auxiliary identities (see Figure 2(a) for an example of an original sequence). Incongruity in these sequences is generally due to a panel depicting the main character’s active imagination, with Calvin imaging himself, others, or objects as animals, monsters/aliens, or other objects (Bimpikou, 2018). In Figure 2(a), panel 1 and 2 show Calvin’s dad sitting in a chair, with Calvin walking up to him. In panel 3, Calvin finishes saying “Kazam,” a typical magic spell, and now there is a big bug sitting in the chair. In panel 4, Calvin walks away, and an off-panel entity asks “What?.” Since a person would be a more likely speaker than a bug, and the (seemingly) calm question implies ignorance of Calvin’s actions, the reader may infer that the dad is actually still in the chair in panel 4, rather than transformed into a bug. Panel 3 then shows how Calvin *imagined* that he changed his dad into a bug with a magic spell, and thus showcases an auxiliary domain (Calvin’s imagination), while panels 1, 2, and 4 belong to the primary domain. The incongruity between figures across panels 2 and 3 can be resolved by inferring that both figures refer to the same identity, i.e., Calvin’s dad. The bug is an auxiliary identity, representing what Calvin imagined his dad looked like for a moment. Twenty of our experimental sequences showed auxiliary identities for characters, as in Figures 1,2, and 4 sequences showed auxiliary identities for objects (where readers should infer that an object within an auxiliary domain refers to an object within the primary domain).

Experimental panels were manipulated so each sequence also had a switch between domains in the reverse direction (compare Figure 2(a–b)) and fully continuous versions Figure 2(c–d), resulting in 96 (24 × 4 conditions) stimuli. In each version, the critical panel (CP) concerned the panel where a second domain was first introduced in the original sequence (either an auxiliary domain Figures 2(a,c) or a primary domain as in Figure 2(b,d). Eight additional comics with no manipulations were added as fillers. The sequences were distributed into four lists according to a Latin Square Design, with each participant viewing 32 comics in total. Each sequence occurred only once in a particular condition, and each condition showcased six different experimental examples.

#### Participants

We recruited participants via a link shared on social media and through the participant pool of Tilburg University, in the Netherlands. The study was approved by the Ethics Committee from the Tilburg School of Humanities and Digital Science (approval code: REC#2018/47B, expiration: October 2021). From a total of 83 participants, in accordance with our preregistration, we randomly selected 60 participants as our sample (38 male, 19 female, 3 other; mean age = 32.05 years, *SD* = 12.80, range = 18–62), who all gave their informed consent for participating. Participants filled in the Visual Language Fluency Index (VLFI) questionnaire, designed to measure expertise with comics (Cohn, 2020a). Scores below 8 reflect low proficiency while scores above 22 reflect a high proficiency, with a score of 12 as average. The current sample had a mean VLFI score of 17.69 (*SD* = 10.99, range = 2.5–44.38). Finally, 70% of participants indicated they were familiar with *Calvin & Hobbes* sequences prior to the experiment.

#### Procedure

The experiment was preregistered (https://aspredicted.org/KWJ_P8S).^1^^1^The preregistration uses slightly different terminology: domain constructions are described as “metaphoric blends” and auxiliary/primary domains as “reality/blended domains.” Since preregistering, these terms have evolved to describe the full extent of this phenomenon (see Klomberg et al. (2023)), besides only a metaphoric angle. Participants accessed the experiment via Qualtrics through an online link, starting with the consent form, instructions, VLFI questionnaire, and a question if they were familiar with *Calvin & Hobbes*. If they were, participants rated how much *Calvin & Hobbes* they read when they were young and currently, on a scale from 1 (my experience is below average) to 5 (my experience is above average). The self-paced viewing experiment used a lab.js JavaScript plugin (Henninger et al., 2019). Prior to each trial, a screen showed the participants’ progress (e.g., trial 1 out of 32). Participants saw each panel of the four-panel sequences one at a time and they proceeded to the next by pressing the spacebar at their own pace. After the final panel, they were asked to rate how comprehensible the entire sequence was by pressing the keyboard (1 = hard to understand, 7 = easy) and how many characters appeared in the sequence (1 to 5), until the 32 sequences were completed. These questions intended to measure how well the sequence was understood and whether participants would count auxiliary identities as novel figures rather than references to existing characters. Participants were then thanked and debriefed, with the final page giving the opportunity to report anything unusual or other additional comments.

#### Data analysis

Viewing times for the critical panels underwent an outlier removal procedure that first excluded viewing times above 10 s, which is far beyond the time needed to read a single comic panel, and below 400 ms, which was deemed too fast to comprehend the image and press to advance (e.g. Foulsham et al., 2016). From this selection, viewing times that were 2.5 SD’s above the mean were removed. If participants had more than 25% of their viewing times removed, they were excluded from analyses. After outlier removal, we had 83 participants since the online survey gathered more responses than anticipated, so we selected the 15 participants first listed in each list to form the sample of 60 participants.

The experiment’s 2 × 2 design included Domain (Domain switch vs. Single domain) and Critical Panel Type (Primary vs. Auxiliary) as within-subjects factors. Using JASP (Version 0.14.1.0; JASP Team 2024), we analyzed viewing times for the critical panel (where the domain switch occurred/would occur), for the subsequent panel, the comprehensibility ratings, and the character ratings using Linear Mixed Effects models, with participants and comics as random effects. We first included our main factors of Domain and Critical Panel Type as fixed effects, and then compared against models also including VLFI scores, to see what better accounted for the variance in the data. We initially also included experience with *Calvin & Hobbes* comics as a factor, averaging across scores for the experience with *Calvin & Hobbes* during childhood and currently. However, this variable correlated with VLFI (*r*(1438) = .46, *p* < .001). Since the latter is a more generally applied measure (Cohn, 2020a), we chose to test only VLFI as an additional fixed factor.

### Results

#### Viewing times

**Figure 3.**
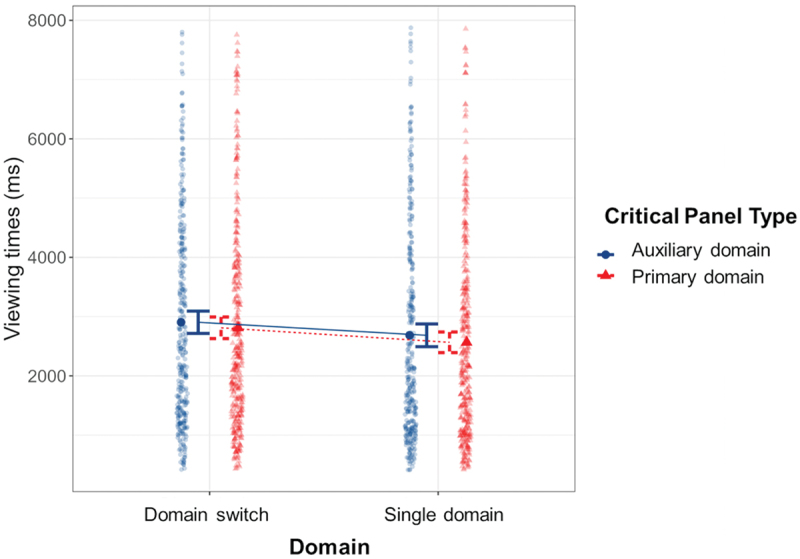
Main effects for domain and critical panel type, with sequences involving a domain switch viewed slower than sequences presenting a single domain, and auxiliary domains viewed slower than primary domains; error bars represent standard errors.

We conducted a Linear Mixed Effects model for the viewing times at the critical panel with Domain and Critical Panel Type as fixed factors first. Here, we expected a main effect of Domain, with domain switches leading to longer viewing times. While our data was not completely normally distributed, Linear Mixed Effects models are sufficiently robust against this violation (Schielzeth et al., 2020). There was a main effect of Domain (*β* = −145.60, SE = 30.22, *p* < .001), with sequences switching between domains (*M* = 2856.24, *SD* = 1746.58; e.g. Figure 2(a–b)) viewed slower than sequences without a switch (*M* = 2624.18, *SD* = 1729.58; e.g. Figure 2(c–d)). Moreover, a main effect of Critical Panel Type (*β* = 94.91, SE = 30.24, *p* = .002) maintain tense that auxiliary domain critical panels (*M* = 2792.74, *SD* = 1778.67; e.g. Figure 2(a,c)) were viewed slower than primary domain ones (*M* = 2689.11, *SD* = 1704.41; e.g. Figure 2(b,d)). Figure 3 illustrates these results.

**Figure 4.**
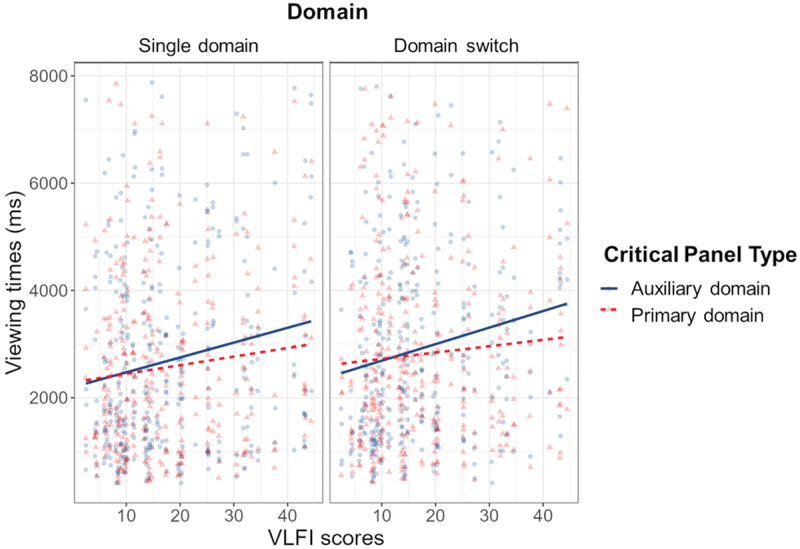
Interaction effect between critical panel type and VLFI, with more experienced comic readers slowing down for auxiliary domain panels.

We compared the first model to one that includes VLFI as a fixed effect. For analyses, we determined the best fit by comparing the two models’ Akaike’s Information Criterion (AIC) and Bayesian Information Criterion (BIC), with the lowest scores showing the best fit. The model including VLFI as a fixed effect produced a slightly better fit (AIC = 23360.10, BIC = 23417.56) when compared to the previous model (AIC = 23381.67, BIC = 23418.24). The model still showed the main effect for Domain (*β* = −138.00, SE = 58.13, *p* = .018), but now a main effect for VLFI emerged (*β* = 26.22, SE = 10.62, *p* = .016), alongside an interaction between VLFI and Critical Panel Type (*β* = 5.91, SE = 2.86, *p* = .039). As illustrated in Figure 4, more experienced comic readers viewed critical panels slower, and more so when these panels showed an auxiliary domain than a primary domain.

Finally, we tested whether the factors affected viewing times of panels following a critical panel, i.e., whether there exists a spill-over effect. Out of our 24 comics, 9 sequences included a panel following a domain switch (in other sequences, the domain switch was the final panel). We fitted a Linear Mixed Effect model for the viewing times of these panels following critical panels with Domain and Critical Panel Type as fixed effects (AIC = 8838.95, BIC = 8868.70) comparing against a model also including VLFI with the fixed effects (AIC = 8819.11, BIC = 8865.86). Across both models, no effects emerged involving Domain or Critical Panel Type, and the model including VLFI appeared a slightly better fit. Here, a main effect again arose for VLFI (*β* = 24.30, SE = 10.34, *p* = .022), indicating that more experienced readers read panels following critical panels slower.

#### Comprehensibility ratings

Our expectation was that sequences with auxiliary identities would be rated as comprehensible. A one sample t-test was conducted to examine whether participants generally considered the sequences comprehensible against the mid-level score of 4 (out of 7). Since the data were not normally distributed, we report the Wilcoxon test. On average, sequences were rated comprehensible (*M =* 5.79, *SD* = 1.45), with the sample median (Mdn = 6) greater than the neutral median of 4, *W* = 834759.00, *p* < .001, *r* = 0.89, representing a large effect. Thus, participants generally understood the comics well.

Next, we fit a Linear Mixed Effects model predicting comprehensibility ratings with Domain and Critical Panel Type as fixed effects (AIC = 4712.13, BIC = 4749.04), and optionally VLFI (AIC = 4747.42, BIC = 4805.42). The base model was the best fit for this data, but included no significant effects, meaning that all sequences were rated equally comprehensible regardless of condition, in line with our expectation. As exploratory analysis, we tested to what extent understanding was affected by processing. We averaged viewing times of the critical panel for each participant for a regression, but this model was not significant (R^2^ = .01, F(1, 58) = 76, *p* = .387).

#### Character ratings

To investigate whether participants perceived auxiliary identities as separate entities, we compared the number of characters that participants perceived to be in the sequence to the number of characters that had to be inferred (i.e., not counting auxiliary identities separately). We created a difference score by subtracting the number of characters to be inferred from the number that was perceived, such that positive values indicate that participants perceived too many identities and negative values indicate that they perceived too few. We omitted 7 sequences for this analysis: 3 sequences that included speech balloons pointing to off-panel characters, making it ambiguous whether this constituted a character *in* the sequence or not, and 4 sequences where the primary and auxiliary identities were objects, and not characters. The analysis included the difference scores for the remaining 17 sequences.

**Figure 5.**
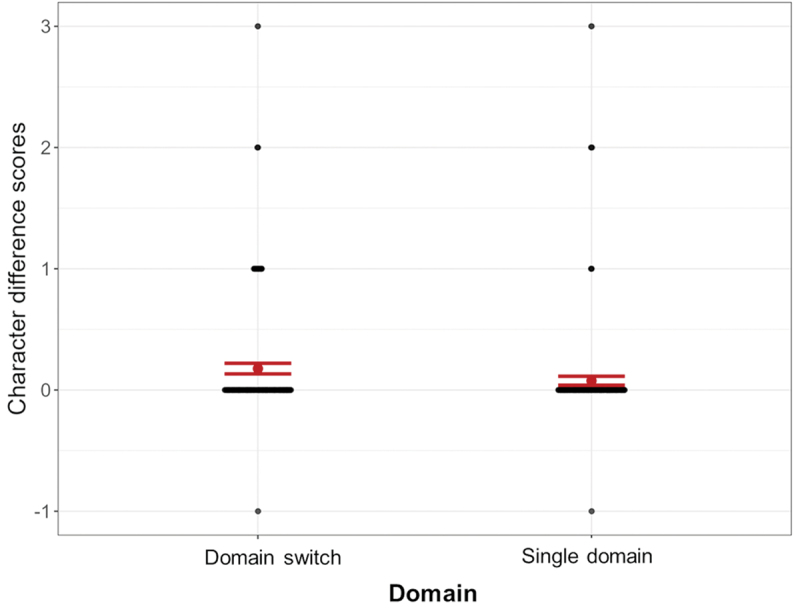
Main effect of domain, with participants perceiving a greater divergence from the correct number of characters for sequences switching between domains than for sequences maintaining a single domain; error bars represent standard errors.

A Linear Mixed Effects model tested this difference score, comparing Domain and Critical Panel Type as fixed factors (AIC = 1014.12, BIC = 1048.61) against a model also including VLFI (AIC = 1063.74, BIC = 1117.94). Both models showed only a main effect of Domain, but we report the statistics from the base model as it had the best fit. The main effect of Domain (*β* = −0.05, SE = 0.01, *p* < .001) indicated that participants perceived more additional characters in sequences switching between domains (*M =*0.18, *SD* = 0.50) than in sequences with a single domain (*M* = 0.08, *SD* = 0.43), as illustrated in Figure 5. However, even for domain switches, 84.31% of difference scores were a 0, meaning that participants mostly did not count auxiliary identities as separate entities; for single domain sequences, this was 94.51%.

### Discussion

Experiment 1 investigated how readers process auxiliary identities in visual sequences. We compared viewing times for sequences with and without domain switches and the types of domains shown. In line with our expectations, readers read panels in sequences with domain switches slower than continuous sequences, and auxiliary domain panels slower than primary domain panels. When including comic reading experience, findings showed more proficient comic readers viewed panels slower, especially when critical panels depicted an auxiliary domain. Despite such differences in processing, no sustained effects emerged for subsequent panels and all sequences were understood equally well. Finally, participants did perceive more characters in sequences with domain switches, though the number of characters was estimated correctly for the majority of sequences.

The longer viewing times for domain constructions with auxiliary identities align with results for other unexpected or inferential narrative techniques in visual storytelling (Brich et al., 2024; Huff et al., 2020; Hutson et al., 2018; Klomberg & Cohn, 2022). These results suggest that domain constructions require more processing to map two storyworld domains and visually distinct agents in a situation model, likely due to the process of incongruity resolution. Critical panels showing auxiliary events were viewed slower in general, even for sequences maintaining an auxiliary domain throughout (i.e., without a domain switch). The depiction of only imagined events thus seems more effortful to process than only primary domain events. One potential limitation is that manipulated sequences with only auxiliary domain events may present greater incongruity with the text than sequences with only primary domain events, e.g. when the text still refers to “Calvin” while the image shows only his auxiliary identity (e.g., an animal). Moreover, these findings imply that the direction of the domain switch is not that significant, as we found no interaction effect for domain switches with a particular type of critical panel domain.

While viewing times differed, these were not related to comprehensibility ratings, and domain constructions were rated just as comprehensible as sequences without such domain switches. This aligns with prior studies on inferential techniques (e.g. replacing a climax moment with an impact star panel or with a panel showing characters reacting to the off-panel climax), which found that inferential sequences were generally comprehensible (Brich et al., 2024; Cohn & Kutas, 2015; Klomberg & Cohn, 2022). Readers thus seem able to understand sequences that require some inferencing. As readers’ responses were not affected by the extended viewing times for domain constructions and auxiliary panels, it suggests that more effortful processing does not necessarily mitigate understanding.

In turn, comic reading experience did relate to viewing times, at least for the critical panel and its subsequent panel. More proficient readers viewed both types of panels longer. Greater proficiency could align with greater attention, potentially due more (intrinsic) motivation for comic reading. Additionally, an interaction emerged between comic reading experience and domain type, with more experienced readers viewing auxiliary domains slower than primary domains. As mentioned before, domain constructions may make sequences more humorous; perhaps proficient readers consider or appreciate the role of a domain switch in the punchline more deeply, contributing to longer viewing times compared to primary domains. In general, proficient readers were relatively slow for subsequent panels regardless of the critical panel content, just as they were relatively slow for the critical panel itself. This could imply that there is no spill-over effect for domain constructions/auxiliary domain panels. Other inferential techniques did often result in sustained effects, which seem to indicate extended processing (Cohn, 2021; Cohn & Kutas, 2015). These findings may suggest that domain constructions are relatively accessible to readers, and that the process of incongruity resolution does not lead to sustained effects.

Auxiliary identities did seem to be perceived as references to primary domain characters, since participants generally responded with the intended number of characters (i.e., counting only primary domain characters). While participants perceived more characters for domain switches than for single domain sequences, the difference appeared slight. The means for both groups were very close to zero, indicating that participants barely over- or underestimated the number of characters. Moreover, for the majority of sequences (domain switches and single domain sequences), participants did not count auxiliary identities as separate entities. It thus seems that participants generally perceive auxiliary identities as references to existing characters, though it may perhaps be slightly more difficult to establish for domain switches than for sequences maintaining a single domain. This means that participants do establish co-reference between distinct appearances, and understood the sequences as expected by theoretical accounts (Klomberg et al., 2024).

## Experiment 2

Experiment 1 showed that readers can resolve auxiliary identities in domain constructions, but with a cost. Experiment 2 further investigates this processing by exploring whether visual features play a role during incongruity resolution. Would particular graphic cues support readers in processing and understanding these sequences? Here, we explore the contours of auxiliary identities across a domain switch. As patterns of structural and graphic cues emerge across domain constructions (Horstkotte & Pedri, 2022; Klomberg et al., 2024; Mikkonen, 2017), we hypothesize that cues like compositional and/or contour similarity across primary and auxiliary entities may facilitate construing cohesion and co-reference for incongruous events and characters.

**Figure 6.**
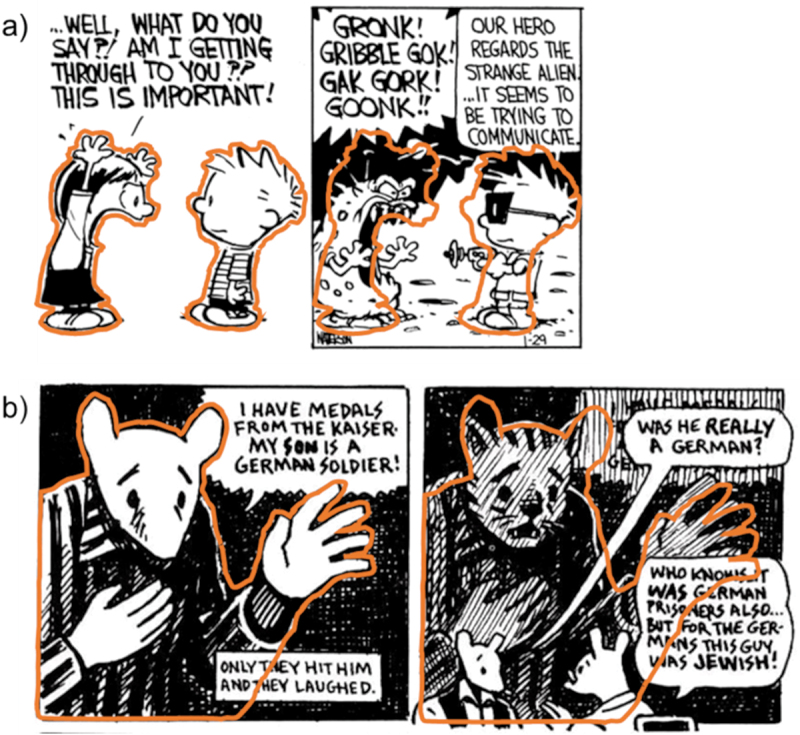
Outline from the first figure copied over the second figure to show contour similarity; figure a slightly adapted from *Calvin & Hobbes* by Bill Watterson © universal press syndicate, figure b slightly adapted from *maus* © art Spiegelman.

In many cases of domain constructions, compositional and/or contour similarity appears between primary domain entities and their auxiliary identities. This is illustrated in Figure 6 which repeats the outlines of characters in the primary domain onto the following panel displaying an auxiliary domain. To show that such similarity is not limited to *Calvin & Hobbes*, consider the sequence from *Maus*, a graphic novel that visualizes Jewish characters as mice and German characters as cats. In Figure 6(b), the father and son at the bottom of panel 2 are discussing the identity of the prisoner shown in panel 1, a character who was shown as a mouse (i.e., Jew) so far, but who claims to be German. In panel 2, the same prisoner is shown behind the father and son, but now with a cat’s head (Pedri, 2013). Given their discussion, and how the character behind them is shaded in, the father and son seem to imagine this prisoner as an actual German person in their minds here, reflecting the possibility that the prisoner might have been speaking the truth (Pedri, 2013). Thus, the prisoner character in panel 2 represents an auxiliary identity, which readers should understand as referring to the prisoner character in panel 1.

Experiment 1 implied that readers can reconcile such lack of co-reference but did not yield insights into how this was achieved. One cue signaling co-reference could thus be compositional and/or contour similarity, which we refer to as *visual rhyme*. Analogous to how verbal rhyme typically stresses overall similarity between words despite a small phonemic difference, the visual rhyme in Figure 6 illustrates similar contours with differences mostly concerning the internal structure of the figures (e.g., different faces).

Such visual likeness across entities has been shown to be beneficial for other narrative techniques that resolve multiple conceptual domains. Namely, similarity in the shape of objects facilitates metaphoric comparisons as compared to objects with dissimilar shape, for both verbal similes combined with pictures (Van Weelden et al., 2014) and visual metaphors (Van Weelden et al., 2011, 2012). Moreover, viewers produce more correspondences between otherwise distinct objects when they were similar in shape (Van Weelden et al., 2011). Seemingly, shape similarity may assist in constructing a meaningful relation across incongruous entities. Therefore, we expect visual rhyme to similarly facilitate processing and understanding of domain constructions that include auxiliary identities, which require inferences of co-reference across incongruous figures.

### Methods

#### Stimuli

**Figure 7.**
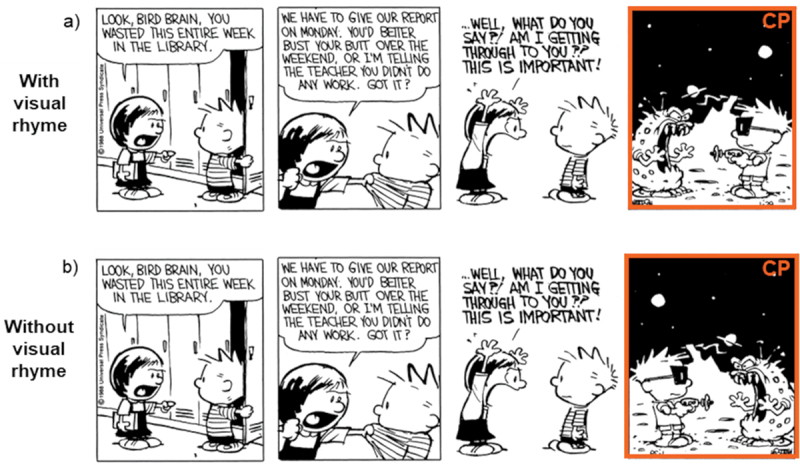
Example stimuli for visual rhyme with wordless critical panels (= CP), showing auxiliary identities a) with visual rhyme and b) without visual rhyme; figures slightly adapted from *Calvin & Hobbes* by Bill Watterson © universal press syndicate.

We selected 20 comics with visual rhyme across primary and auxiliary domain characters from *Calvin & Hobbes*, which were not used in Experiment 1. Our factor of Visual Rhyme included two levels: critical panels in their original form (i.e., With visual rhyme, see Figure 7(a)) and critical panels where rhyming shapes were horizontally flipped, disrupting contour similarity (i.e., Without visual rhyme panels, see Figure 7(b)). Since Experiments 1 and 2 ran at the same time and required an equal number of trials, the same eight filler comics without manipulations from Experiment 1 were joined by four other filler comics to reach a total of 32 comics. The sequences were distributed into two lists according to a Latin Square Design. Each participant viewed 32 sequences in total, with each experimental sequence shown once in a particular condition, and with each condition including ten experimental examples.

A first analysis of this dataset indicated that the presence of words was a confound for viewing times. This sample included 37 participants (9 male, 22 female, 6 others) with a mean age of 25.16 (*SD* = 8.27, range = 18–49). Viewing times did not differ for conditions, *F*(1, 632.03) = 1.02, *p* = .313, but a moderate correlation emerged between viewing times and the amount of words at the critical panel, *r*(688) = .43, *p* < .001, with more words predicting longer viewing times. This suggests that viewing times were affected by the number of words rather than the experimental conditions. Therefore, we designed a new experiment using the same stimuli in which all words in the experimental critical panel were omitted (from here on referred to as Experiment 2), as shown in Figure 7

#### Participants

Experiment 2 was approved by the same Ethics Committee as Experiment 1 under the same code, and shared via the same channels, resulting in 34 participants (19 male, 15 female) with a mean age of 28.74 (*SD* = 10.5, range = 18–57). All participants gave their informed consent. The average VLFI score was 18.56 (*SD* = 9.94, range = 1.88–48.75). Here too, most participants (60%) were familiar with *Calvin & Hobbes* prior to the experiment.

#### Procedure

This experiment was also preregistered (https://aspredicted.org/BDQ_GC4).^2^^2^The preregistration again uses some outdated terminology, e.g. “alternative domain” instead of auxiliary domain. The remaining procedure was the same as in Experiment 1.

#### Data analysis

We applied the same outlier removal procedure that excluded viewing times of critical panels above 10 s or below 400 ms, and then viewing times above the mean plus 2.5 times the SD. We also omitted participants that had more than 25% of their viewing times removed. We fit Linear Mixed Effects models for viewing times at the critical panel and comprehensibility ratings for the whole sequence, again with participants and comics as random effects. We compared models with Visual Rhyme as a fixed effect against models that also included VLFI scores. As VLFI scores correlated strongly with participants’ experience with *Calvin & Hobbes* (*r*(678) = .53, *p* < .001), we again only tested VLFI.

### Results

#### Viewing times

Our data were again not normally distributed, but Linear Mixed Effects models have been shown to handle such violations (Schielzeth et al., 2020). We expected visual rhyme to lead to faster viewing times, but for the base model including Visual Rhyme as a fixed effect (AIC = 11106.81, BIC = 11129.37) no effects emerged, indicating similar viewing times for rhyming and non-rhyming panels.

**Figure 8.**
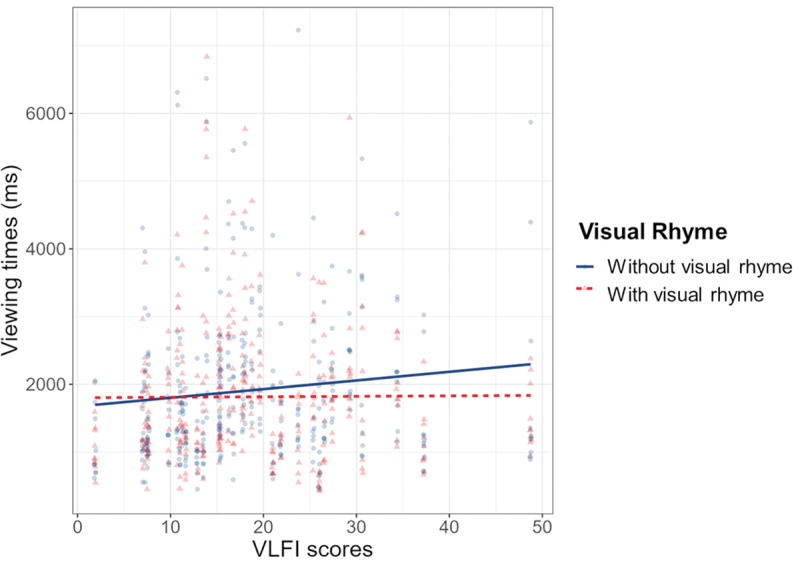
Interaction effect between visual rhyme and VLFI, with more experienced comic readers slowing down for panels without visual rhyme.

The model including VLFI as additional fixed effect appeared a slightly better fit for this data (AIC = 11095.45, BIC = 11127.04), and showed an interaction effect for VLFI and Visual Rhyme (*β* = 6.98, SE = 3.44, *p* = .043). Estimated trends indicated that for panels without visual rhyme, higher proficiency led to slower viewing times (*β* = 13.96, SE = 6.87, *p* = .042), as illustrated in Figure 8 Thus, the more experienced comic readers are, the longer they viewed panels without visual rhyme.

#### Comprehensibility ratings

A one-sample t-test again compared comprehensibility ratings to the mid-level comprehensibility score of 4 (out of 7). Since the ratings were not normally distributed, a Wilcoxon test was used, which showed that comics were understood well (*M* = 5.66, SD = 1.50), and that their ratings (*Mdn* = 6) were greater than the neutral median of 4, W = 176468.00, *p* < .001, *r* = 0.87, with a large effect.

**Figure 9.**
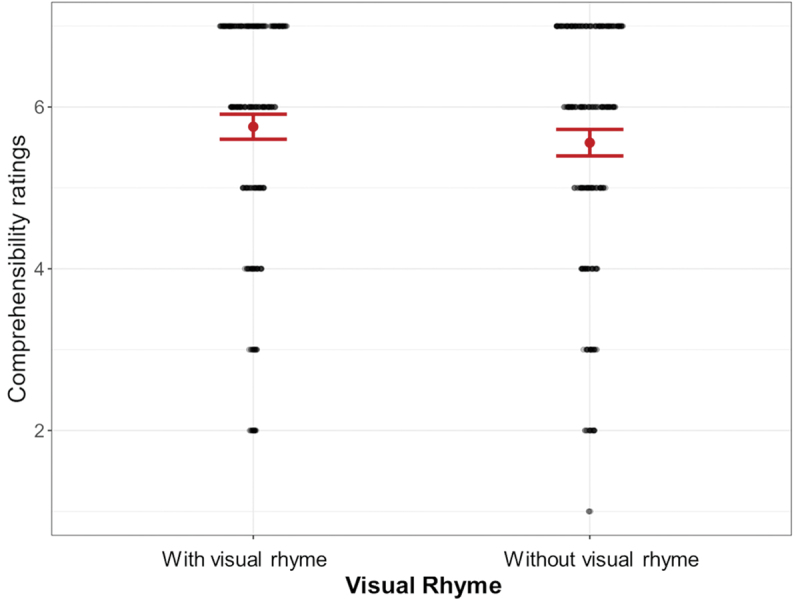
Comprehensibility ratings for sequences including panels with and without visual rhyme, with rhyming panels rated more comprehensible; error bars represent standard errors.

These ratings were then analyzed with a Linear Mixed Effects model predicting comprehensibility ratings with Visual Rhyme (AIC = 2270.33, BIC = 2292.94) and optionally VLFI as additional fixed effect (AIC = 2288.89, BIC = 2320.55). Here, we expected visual rhyme to lead to higher comprehensibility ratings. Since the base model provided the best fit, we report those results. A main effect for Visual Rhyme (*β* = −0.10, SE = 0.05, *p* = .033) indicated that sequences with visual rhyme (*M* = 5.76, *SD* = 1.45) were rated more comprehensible than sequences without visual rhyme (*M* = 5.56, *SD* = 1.54), as shown in Figure 9

Finally, we again conducted an exploratory regression to investigate the impact of processing on comprehensibility. We averaged viewing times of the critical panel for each participant, but here too, the model showed no effects (R^2^ = 0.03, *F*(1, 32) = 0.09, *p* = .763).

#### Character ratings

We again omitted 2 sequences where speech balloons pointed to off-panel characters; the remaining 18 sequences all switched between primary and auxiliary characters (not objects). With again a difference score (= number of perceived characters – number of characters to be inferred) as dependent variable, we conducted a Linear Mixed Effects model to test whether participants counted auxiliary identities as separate characters. The best fit for our data was the base model including Visual Rhyme as a fixed effect (AIC = 1258.86, BIC = 1280.95), rather than also including VLFI (AIC = 1279.90, BIC = 1310.82). No main effect emerged, indicating that the orientation of the critical panel did not affect how many characters participants counted. Across both rhyming and non-rhyming panels, around 66–67% of difference scores were 0 (meaning that auxiliary identities were not counted separately). Around 19–21% of difference scores were 1, indicating that participants perceived 1 character too many.

### Discussion

Experiment 2 explored the effect of visually similar contours across primary and auxiliary entities on the viewing times for sequences with domain constructions. Visual rhyme did not facilitate processing directly, but panels without rhyming contours were viewed longer by readers with high comic reading proficiency, while panels with visual rhyme were generally rated as more understandable than panels without rhyme. Moreover, most times readers correctly estimated the number of characters to be inferred in the sequence.

While the results indicate that visual rhyme did not affect processing directly, sequences with visual rhyme appeared more comprehensible to readers. The viewing times may then suggest that perhaps a *lack* of rhyme hindered more experienced comic readers, as non-rhyming panels were viewed longer by readers with higher VLFI-scores (i.e., more proficient comic readers). Visual rhyme sequences were all original, existing sequences, which could imply that such rhyme is an established cue. Disturbance of this cue could pose challenges to readers more exposed to comics who may expect it for such inferential sequences. Readers who are less proficient comic readers then would not have such expectations, which would explain their similar viewing times for panel both with and without rhyme. If indeed visual rhyme across primary characters and their auxiliary identities is an established cue to signal a (co-referential) relation between the two characters, this would align with theoretical observations that graphic cues can support inferences of meaning for domain constructions (Horstkotte & Pedri, 2022; Klomberg et al., 2024).

One potential reason that we did not find the expected results of visual rhyme facilitating both viewing times and comprehensibility may be that our manipulation of contour similarity also (inadvertently) confounded with the 180° rule. This rule refers to how filmmakers often maintain a sense of coherence between shots by showing shots from one “side” of the scene; in other words, an imaginary line divides the scene in two, and the camera is placed alongside a 180° semicircle on one side of this line (Baker & Levin, 2015; Huff & Schwan, 2012; Kraft et al., 1991). Prior work on violations of the 180° rule in images found that viewers were less accurate in reconstructing spatial scenes when the original left/right orientation of images had been flipped (Kraft et al., 1991), and viewers struggled with recognizing the original image’s orientation more for those violations (Kraft & Jenkins, 1977; Kraft et al., 1991). For video sequences, viewers preferred to interpret ambiguous spatial sequences as maintaining the 180° rule, and they were slower to respond for non-ambiguous sequences that violated this rule (Huff & Schwan, 2012). The 180° rule violations also functioned as a cue for viewers to perceive a new event, which prompted them to compare features of the current scene to features held in working memory (Baker & Levin, 2015). Thus, this violation may lead to prolonged processing and confusion about spatial structure and event structure. Within our study, we cannot rule out that panels without visual rhyme were rated less comprehensible than rhyming panels because the former violated the established composition, and were therefore experienced as a less coherent sequence.

If indeed lower comprehensibility ratings would be due to the violation of the 180° rule, it remains an open question why this did not affect viewing times consistently across readers. Readers overall noticed our manipulation and found it less coherent (as indicated by the ratings), but only more experienced readers spent more time on these panels. Less experienced readers might view difficult sequences less attentively or spend less effort on investigating odd panels, while more experienced readers were driven to reconcile the violated spatial structure with the representation stored in their mind, seeking an explanation to restore coherence. This could even go in tandem with the idea that experienced readers expected visual rhyme across domain switches and struggled when this cue is missing. At the least, our findings showed that non-rhyming panels were less comprehensible, though the manipulation affected processing only for readers familiar with comics. To further study the effects of visual rhyming on incongruity resolution, future research could construct stimuli that affect contour similarity but honor the 180° rule.

Visual Rhyme did not affect how many characters readers perceived in the sequence. Even though a greater proportion of readers now estimated there to be 1 character too many compared to Experiment 1, most sequences were still interpreted correctly. Moreover, while the presentation of the critical panel does not affect such perceptions, domain switches did. The exact positioning of figures thus does not seem to confuse readers with regard to the number of characters, while mentally reconciling figures as co-referential did have such effect. Overall, readers still successfully resolved auxiliary identities.

## Experiment 3

**Figure 10.**
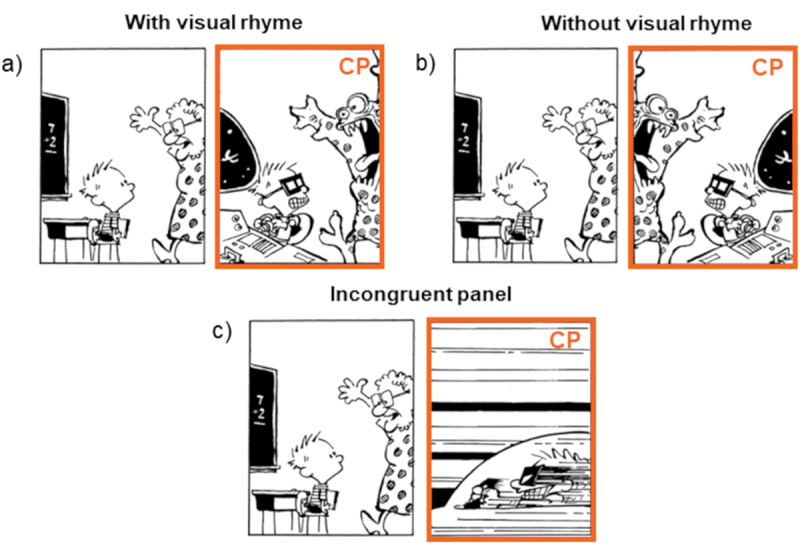
Example stimuli for the panel match factor, showing panel pairs that depict their auxiliary identities a) with visual rhyme, b) without visual rhyme, and c) a panel incongruent with the narrative; figures slightly adapted from *Calvin & Hobbes* by Bill Watterson © universal press syndicate.

To further explore the effect of visual rhyme, our third experiment investigated to what extent the *strength* of visual rhyme affects processing. By considering how weak or strong visual rhyme affects results, we provide more nuance to the findings of Experiment 2, where we considered only the presence or absence of contour similarity. We again measured viewing times, but also asked participants to rate the perceived visual similarity across panel pairs, as shown in Figure 10 These ratings constituted a “Rhyme Measure,” with high scores indicating strong visual similarity in contours, i.e., strong visual rhyme, and low scores indicating weak visual similarity/rhyme. We then tested these ratings of visual rhyme (the Rhyme Measure) as a fixed effect for the viewing times sampled in Experiment 2 and 3, and the comprehensibility ratings sampled in Experiment 2. In other words, we tested to what extent this Rhyme Measure predicted viewing times and/or comprehensibility ratings. In line with Experiment 2’s findings, we expected weak rhyme (low visual similarity scores) to correlate positively with slower reading times, while stronger visual rhyme (high similarity scores) would correlate positively with comprehensibility ratings.

### Methods

#### Stimuli

We selected 45 panel pairs in total. We chose 36 panel pairs from Experiments 1 and 2 that appeared to have similar contours across characters from primary and auxiliary domains. Sixteen panel pairs came from Experiment 1, 20 from Experiment 2 (all its stimuli), and 9 pairs were novel to this experiment. The factor Panel Match included Experiment 2’s conditions (With visual rhyme vs. Without visual rhyme) and a third condition showing a panel from an unrelated sequence. As in Experiment 2, the panel pairs’ original form was with visual rhyme, and critical panels were horizontally flipped to create a panel condition without visual rhyme (see Figure 10(a-b)). Here, the critical panel refers to the second panel of each pair, as that panel introduced a second domain (which could either be a primary or auxiliary domain). Finally, an incongruent panel condition presented panels from unrelated sequences that visually had little to no rhyme and no obvious meaningful relation with the preceding panel (Figure 10(c)). This incongruous panel showed the same domain type as the other conditions (e.g., in Figure 10(c)), also an auxiliary domain showing imagination). Each participant viewed all 45 panel pairs in total, which occurred only once in a particular condition, with each condition having 15 examples.

#### Participants

The experiment was approved by the same Ethics Committee as Experiment 1 and 2 under the same approval code. Sixty-one participants were recruited via the participant pool of Tilburg University (23 male, 38 female; mean age = 21.03, *SD* = 2.86, range = 18–30). The average VLFI score was 12.42 (*SD* = 6.51, range = 3.75–36.63). Only 19.67% of participants indicated they knew *Calvin & Hobbes* prior to the experiment.

#### Procedure

Participants entered a randomized battery of four experiments in a lab environment, all run in Psychopy (Peirce et al., 2019), one of which was Experiment 3. Prior to the experiment on screen, they filled out a consent form and the VLFI questionnaire, after which the self-paced reading experiment on screen began. It started with an explanation of visual rhyme, with an example showing visual rhyme across panels (i.e., Calvin and his environment having similar contours to a spaceman and his spaceship) and an example with semantic coherence but no visual rhyme (i.e., Calvin doing a same action as an animal, while having distinct contours), to emphasize that participants should focus on (dis)similarity in graphic lines across panels rather than on meaning. Next, a screen with instructions explained that they would see the panel pairs one image at a time, after which they would press the space bar to continue to the next image, and then would rate the visual similarity between the two images (1 = no visual similarity, 7 = strong visual similarity). Once all trials were completed, participants were debriefed and thanked for their participation.

#### Data analysis

Viewing times for critical panels were verified with the same procedure as previous experiments, excluding those above 10 s, below 400 ms, and those within that selection that were above the mean plus 2.5 times the SD. We also removed participants with more than 25% of viewing times missing. Remaining viewing times and the perceived similarity ratings were analyzed with Linear Mixed Effects models, with participants and comics again as random effects, and Panel Match as a fixed effect, comparing against models including also VLFI as a fixed effect to estimate the best fit.

In addition to these Linear Mixed Effects models, we also included the responses to the perceived similarity ratings (the Rhyme Measure) as a fixed effect, testing to what extent visual rhyme between the critical panel and its preceding panel affected processing times (Experiment 2, 3) and comprehensibility ratings (Experiment 2). Experiment 1 had too few matching panel pairs with Experiment 3 for the Rhyme Measure to be applied, as we manipulated those sequences to have distinct preceding panels. We first present the results of the Rhyme Measure for the current experiment and then its application to Experiment 2.

### Results

#### Viewing times

**Figure 11.**
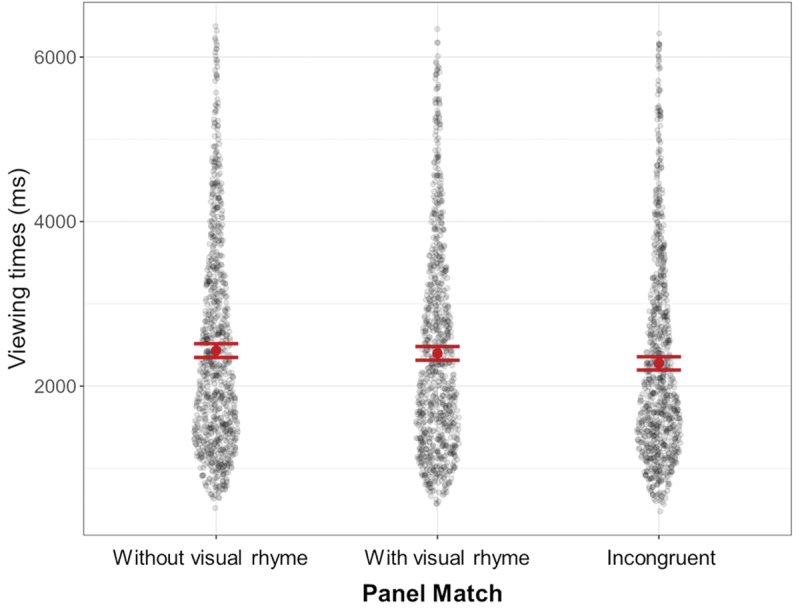
Main effect of panel match, with incongruent panels viewed faster than rhyming and non-rhyming panels.

Data was again not normally distributed, but, as indicated, Linear Mixed Effects models are fairly robust against this violation (Schielzeth et al., 2020). We compared a Linear Mixed Effects model with Panel Match as a fixed effect (AIC = 44308.10, BIC = 44343.38) against one also including VLFI (AIC = 44293. 42, BIC = 44346.33). We had no prior hypothesis for contrasts in viewing times between panels themselves (incongruent and with/without visual rhyme). As the model with VLFI was only a better fit for AIC, we report the base model, where a main effect of Panel Match emerged. Estimated marginal means indicated a contrast between incongruent panels and rhyming panels (*β* = 135.24, SE = 48.78, *p* = .011), and between incongruent panels and non-rhyming panels (*β* = 155.28, SE = 48.61, *p* = .004). Incongruent panels (*M* = 2276.13, *SD* = 1223.02) were viewed faster than panels with visual rhyme (*M* = 2397.86, *SD* = 1255.20) and faster than panels without visual rhyme (*M* = 2432.27, *SD* = 1266.24). Rhyming and non-rhyming panels did not differ from one another (*β* = 20.05, SE = 48.79, *p* = .681; see also Figure 11).

#### Perceived similarity ratings

**Figure 12.**
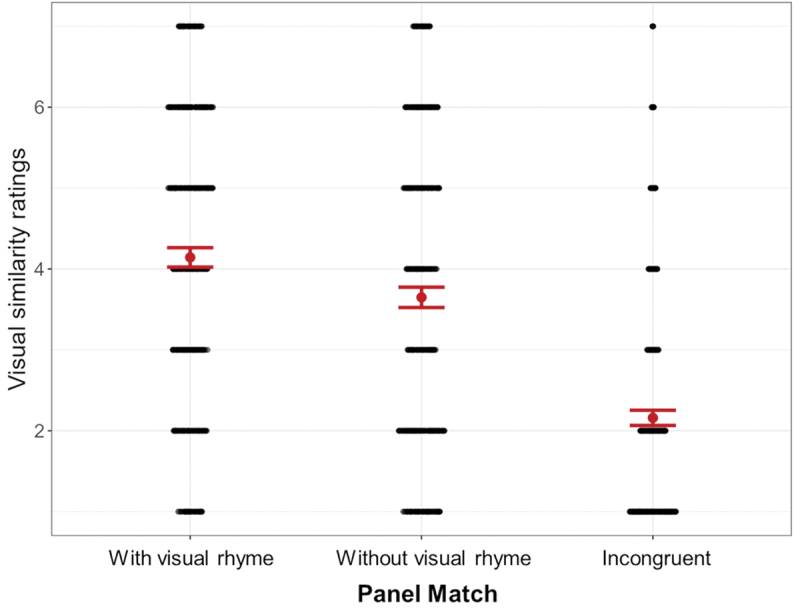
Main effect for panel match, with rhyming panels rated more visually similar than non-rhyming panels, which were again more similar than incongruent panels; error bars represent standard errors.

To see whether panel pairs were rated differently, we fit a Linear Mixed Effects model to the perceived similarity ratings, comparing Panel Match as a fixed effect (AIC = 10141.48, BIC = 10176.99) against a model with also VLFI (AIC = 10170.44, BIC = 10223.69). The base model appeared the best fit and indicated a main effect. Estimated marginal means showed a contrast between panels with visual rhyme and without (*β* = 0.50, SE = 0.07, *p* < .001), between panels with visual rhyme and incongruent panels (*β* = 1.99, SE = 0.07, *p* < .001), and between panels without visual rhyme and incongruent panels (*β* = 1.49, SE = 0.07, *p* < .001). Panels with visual rhyme (*M* = 4.14, *SD* = 1.85) were judged more visually similar than the other two panel types, and panels without visual rhyme (*M* = 3.65, *SD* = 1.94) were judged more similar than incongruent panels (*M* = 2.16, *SD* = 1.45), as illustrated in Figure 12

#### Rhyme measure applied to current results

**Figure 13.**
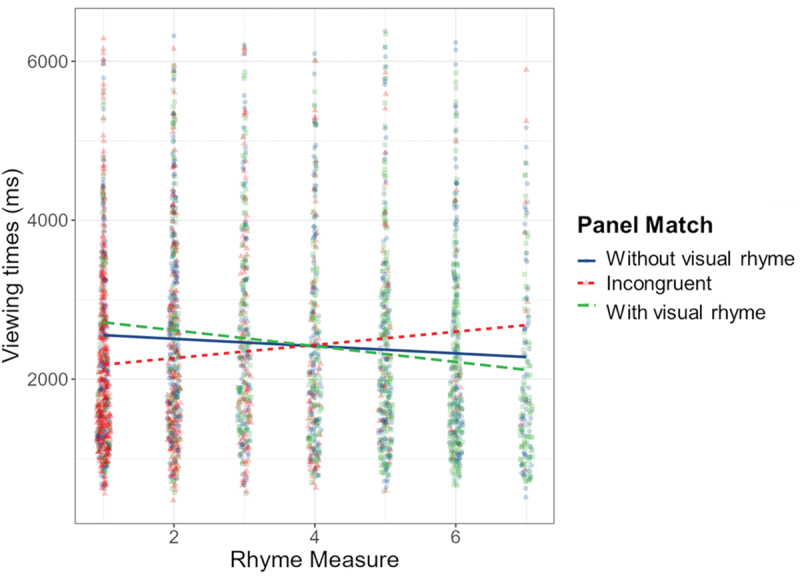
Interaction effect for panel match and rhyme measure, with ratings of greater visual similarity predicting faster viewing times for visually rhyming and non-rhyming panels, but slower viewing times for incongruent panels.

Since participants did perceive differences in visual similarity across conditions, we investigated to what extent this rhyme measure might predict viewing times. The hypothesis was that weak rhyme would lead to slower reading times (in line with Experiment 2’s findings). We therefore tested Rhyme Measure (the visual similarity ratings) as a fixed effect alongside Panel Match (AIC = 44249.82, BIC = 44302.73), and this model appeared a better fit than the other models testing viewing times at the critical panel. Alongside the main effect of Panel Match, an interaction between Panel Match and Rhyme Measure emerged. Estimated trends showed the same contrasts as for viewing times, namely between incongruent and rhyming panels (*β* = −192.86, SE = 31.56, *p* < .001) and between incongruent panels and non-rhyming panels (*β* = −169.26, SE 30.71, *p* < .001), with no contrast between rhyming and non-rhyming panels (*β* = 23.56, SE = 25.91, *p* = .363). As shown in Figure 13, greater visual similarity aligns with faster viewing times for panels with and without visual rhyme, while for incongruent panels, it results in slower viewing times.

#### Rhyme measure applied to experiment 2

To examine how visual rhyme affects processing for sequences, we tested the Rhyme Measure as predictor for the viewing times in Experiment 2. As before, we expected weak rhyme to align with slower reading times, based on Experiment 2’s own results. We averaged the similarity ratings of panels with and without visual rhyme for each item to constitute the Rhyme Measure there, since all critical panels from Experiment 2 were rated during Experiment 3.

**Figure 14.**
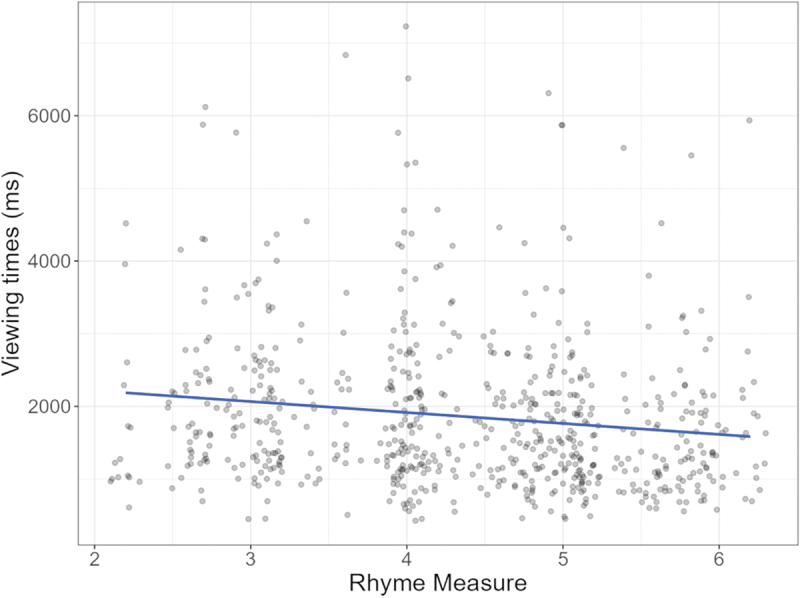
Main effect of rhyme measure, with greater graphical similarity predicting faster viewing times.

We conducted a Linear Mixed Effects model with participants and comics as random effects, and Visual Rhyme and Rhyme Measure as fixed effects. This model appeared to have better fit than prior models (namely, AIC = 11087.56, BIC = 11119. 15) and showed a main effect of Rhyme Measure (*β* = −115.66, SE = 57.44, *p* = .049). As can be seen in Figure 14, sequences with greater visual similarity were viewed faster.

Finally, to test whether the strength of visual similarity affected understanding, we fit a Linear Mixed Effects model to the comprehensibility ratings from Experiment 2, with Rhyme Measure as a fixed effect alongside Visual Rhyme. While we expected strong visual rhyme to lead to higher comprehensibility ratings, this model appeared a worse fit (AIC = 2279.64, BIC = 2311.29) than the model with just Visual Rhyme, and no effects emerged.

### Discussion

Experiment 3 tested whether the strength of perceived visual rhyme across panels affected processing and comprehensibility of auxiliary identities. We used participants’ visual similarity scores, expecting that weak visual rhyme would lead to slower viewing times and strong visual rhyme to greater comprehensibility ratings. Rhyming panels were recognized as more visually similar than non-rhyming and incongruent panels, and non-rhyming more similar than incongruent panels. An interaction between Panel Match and visual similarity scores showed that viewing times were faster for panels with higher Rhyme Measure scores, at least for the panels that were meaningfully related to the prior panel (i.e., panels with and without visual rhyme). This finding was replicated using Experiment 2’s results, where the Rhyme Measure predicted faster viewing times for more visually similar panels. Contrary to expectations, the Rhyme Measure did not affect understanding.

Experiment 3 substantiated that readers differentiate visual similarity across panels showing auxiliary identities, with visual rhyme panels deemed most visually similar, followed by panels without visual rhyme, and then incongruent ones. For incongruent panels, greater visual similarity seemed to lead to slower viewing times, which could potentially be explained by readers spending more time to integrate unrelated events for panels that were more visually similar. This speculation aligns with our proposal that visual similarity across panels typically signals semantically related characters and/or domain constructions that can be resolved cohesively (Klomberg et al., 2023, 2024). However, it should be taken into account that incongruent panels were generally rated as having weak visual rhyme, and a lack of data for higher visual similarity scores warrants care in interpreting these results.

The effect of visual rhyme manifests most convincingly when this measure was used to predict viewing times sampled in Experiment 2. Here, readers were not asked explicitly to rate visual similarity and focused predominantly on understanding a complete sequence. Visual similarity ratings significantly predicted faster viewing times but had no effect on understanding. This suggests that graphic cues affect online viewing processes but do not so much affect long-term meaning storage, given that details like lateral composition are not stored, while meaning is retained (Gernsbacher, 1985). Overall, these results support that stronger visual rhyme across domain switches facilitates processing for readers.

## General discussion

This project investigated the processing and understanding of auxiliary identities in domain constructions. An auxiliary identity is a character from an auxiliary domain (i.e., events understood as mental experiences) that is meant to refer to a character from a primary domain (i.e., the physical, “real” storyworld). Despite different appearances, readers need to construe co-reference between these two characters (the primary and auxiliary version) and understand the change in appearance is due to a character dreaming, imagining, or engaging in another type of auxiliary domain event. In Experiment 1, we found that sequences with such auxiliary identities took more time to process but were rated as understandable as sequences without domain switches/auxiliary panels. Experiment 2 found that panels that switched to another domain without visual rhyme led to longer viewing times for proficient comic readers, while panels with visual rhyme were rated as more understandable. Experiment 3 substantiated that domain switches were processed faster when panels exhibited strong rhyme. Across experiments, greater experience with comic reading led to slower viewing times.

The findings indicate that domain constructions with auxiliary identities require extended updating processes but do not affect understanding. In Experiment 1, longer processing occurred for domain switches without costs to comprehensibility, and in Experiment 3, greater visual similarity predicted faster processing but not greater understanding. This suggests that updating costs do not directly affect readers’ understanding of the sequence, in contrast to previous assumptions for inferential techniques (Huff et al., 2020; Klomberg & Cohn, 2022). Alternatively, the findings could suggest that greater effort is spent to reach the same comprehensibility ratings. The counts for characters indicated that readers by and large perceived the correct number of characters, which implies that readers were successful in integrating distinct domains and their respective agents, whether despite *or* because of extended processing.

In addition, we found that only the orientation of the panel affected understanding, with panel pairs with visual rhyme producing higher comprehensibility ratings than panel pairs without visual rhyme. However, since the Rhyme Measure did not predict comprehension in a similar way for Experiment 2’s ratings, this may suggest that the non-rhyming panel condition, where the panel was flipped horizontally to disrupt the rhyme, was simply more disruptive for readers and therefore considered less cohesive, as in 180° rule violations (Baker & Levin, 2015; Huff & Schwan, 2012; Kraft et al., 1991) and other more discontinuous information units in visual narratives (Klomberg & Cohn, 2022; Loschky et al., 2020; Manfredi et al., 2017). Future research can investigate non-rhyming panels that maintain the 180° rule, to explore to what extent this rule affects comprehensibility of a sequence.

While visual rhyme had no effect on self-reported comprehensibility, this graphic cue does facilitate the processing of auxiliary identities. Higher visual similarity scores correlated with faster viewing times in Experiment 2 and 3. Moreover, the results from Experiment 2 also implied that experienced readers viewed non-rhyming compositions slower than rhyming compositions. This aligns with prior research suggesting that greater similarity motivates readers to find semantic connections (Van Weelden et al., 2011). Potentially, such graphic cues were missing or less conventionalized for other inferential techniques, which were rated less comprehensible that the explicit depiction of events (Cohn & Kutas, 2015; Klomberg & Cohn, 2022). Future work into visual narratives is recommended to verify strength of visual rhyme across panels, and further explore how these cues interface with interpretations of meaning. Moreover, other graphic and structural cues (see e.g. Klomberg et al., 2024) should be tested, to further investigate to what extent formal features affect processing. Here, visual rhyme indeed appeared beneficial for processing domain constructions with auxiliary identities.

Differences between readers may also affect how they process auxiliary identities, as comic reading experience affected viewing times throughout experiments. More experienced readers were slower for panels showing an auxiliary domain (Exp. 1) and for mirrored panels (Exp. 2). Moreover, comic reading experience predicted slower reading in general. Such findings may imply that more proficient readers make deeper connections in establishing situation models, or, alternatively, that less proficiency may cause readers to gloss over complex sequences sooner. Since proficiency did not affect understanding here, this remains a question for future research.

Furthermore, greater exposure to comics may lead readers to encode graphic cues as patterns in visual storytelling. The fact that the visually rhyming sequences used in our experiments were all original, unmanipulated compositions, and that such visual similarity across domain switches has been observed in numerous visual narratives (Horstkotte & Pedri, 2022; Klomberg et al., 2024; Mikkonen, 2017), warrants the idea that visual rhyme is a conventionalized technique employed by comic artists to cue readers in construing co-reference across primary and auxiliary identities. This would explain why in Experiment 2 more proficient readers slowed down for panels that disrupt visual rhyme: the more readers engage with visual narratives, the more likely they are to store such cues in their long-term memory and rely on them during processing. There seems to be sufficient opportunity for readers to encounter domain constructions in published comics, with prior corpus work indicating that around 65% of comics (27 in a corpus of 40 total) included an auxiliary domain event (van der Gouw, 2022).

The comprehensibility and frequency of domain constructions, including the role of visual composition and graphic cues, challenge assumptions that visual narratives are understood only through processes of scene perception or event cognition (Loschky et al., 2020) or that they negotiate visual co-reference through purely pragmatic (Abusch, 2012; Maier & Bimpikou, 2019; Schlöder & Altshuler, 2023) or perceptual (Gestalt) principles (Bateman & Wildfeuer, 2014; Gavaler & Beavers, 2018). These incongruities found in domain constructions do not arise in everyday visual perception, and proficiency could be relevant for their construal. This aligns with empirical findings showing that comprehenders unfamiliar with visual narratives may fail to construe co-reference across images, even if they recognize the similarity across objects (for review, see Cohn, 2020a). Visual co-reference appears based on a range of basic principles that must be learned through exposure with visual language, and auxiliary identities further extend such encoded patterns (Klomberg et al., 2023). These patterns would be learned through experience with visual narratives, which could be investigated by comparing our samples with above-average comic reading proficiency to samples with lower proficiency.

The kind of domain constructions studied here align with a broader set of inferential techniques that replace key events in visual narratives, which also have been shown to require extended processing but without hindering an overall understanding of the sequence (Brich et al., 2024; Cohn & Kutas, 2015; Hutson et al., 2018; Klomberg & Cohn, 2022; Magliano et al., 2017). Such bridging inferencing occurs across modalities and has been shown to be resolved quickly and efficiently by readers/viewers (Papenmeier et al., 2019; St.; George et al., 1997; Strickland & Keil, 2011). Prior work has also showed that readers enjoy sequences where incongruity could be resolved meaningfully, and find them aesthetically appealing (Giora et al., 2004; Ramachandran & Hirstein, 1999). Furthermore, domain constructions connect to research on multiple narrative worlds embedded within stories (e.g. *The Neverending Story*), which are common in narratives and inform about processes underlying narrative comprehension (Chan et al., 2018). These connections illustrate the wide range and aesthetic appeal of inferential techniques in visual storytelling, which domain constructions would also be part of.

In conclusion, this work investigated cognitive processes related to auxiliary identities, where distinct appearances can refer to a single character because one appearance originates from an auxiliary domain, i.e., a (private) mental experience (a dream, memory, imagination, etc.). Our findings support that readers understand these constructions well and as intended, despite extended processing, and that such processing seems facilitated by graphic cues within the narrative. Such an interface between form and meaning could be used as a patterned cue across narratives, encoded by more proficient readers. This work thus highlights specific affordances of the visual modality that connect to conceptual perspective-taking and (co-referential) inferencing within visual narratives. Overall, it supports and further develops theoretical studies from fields of discourse (Abusch & Rooth, 2022; Maier & Bimpikou, 2019), focalization (Horstkotte & Pedri, 2022), perspective taking (Mikkonen, 2017), and visual language theory (Klomberg et al., 2024) on the understanding of narrative constructions in storytelling.
